# Risk preference and rural livelihood transitions in the hilly and mountainous region of southern China: a case study in Ruijin City

**DOI:** 10.1038/s41598-024-77356-z

**Published:** 2024-10-26

**Authors:** Zhilong Wu, Hao Chen, Tian Zeng, Yushan Yan, Mengyuan Zhang

**Affiliations:** 1 College of Applied Economics, Jiangxi University of Finance and Economics, No. 169, East Shuanggang Road, Jingkai District, Nanchang, 330013 China; 2 Institute of Ecological Civilization, Jiangxi University of Finance and Economics, Nanchang, 330013 China; 3 Jiangxi University of Applied Science, Nanchang, 330100 China

**Keywords:** Risk preference, Rural livelihood transition, Non-agriculturalization, Non-grainization, Anti-urbanization, Hilly and mountainous area, Ecology, Environmental social sciences

## Abstract

Rural livelihood transition towards non-agriculturalization, non-grainization and even anti-urbanization has become a thorny social problem that undermines farmland resources and worldwide food security. Based on a simulation survey, this study explored the risk preferences and the livelihood transition mechanisms of typical farmers in the hilly and mountainous region. The results indicated that: (1) 76.86% of rural households exhibited risk aversion tendencies, with 60.67% being highly risk-averse. The ranking of risk aversion among the three typical farmers is consistent with asset abundance, with non-agriculture oriented households > semi-farmer and semi-labour households > vocational farmer households. (2) The non-grainization of vocational farmer households is significantly and positively correlated with the family labour force, land management area, and housing assets, yet negatively correlated with risk preferences. Compared to traditional grain cultivation, non-grainization in the hilly and mountainous region possesses lower risks and higher profitability for vocational farmer households. (3) The total non-agriculturalization of semi-farmer and semi-labour households correlates negatively with land management area but positively with family income. (4) Anti-urbanization and returning hometowns for farming are still regarded as a livelihood fallback by the non-agriculture oriented households, but excessive gift expenditure has become a heavy burden in rural society. Therefore, practical and systematical countermeasures are proposed in this research to guide sustainable livelihood transition.

## Introduction

In the context of urbanization and industrialization, rural livelihood transition towards “non-agriculturalization” and “non-grainization” has become a main trend in developing China. As the consequence, it brought forth large labour migration from rural to urban areas^1^, sharp decrease in farmers’ willingness to grow crops^2^, pervasive abandonment of cultivated land^3^and residential land^4^, and posed severe challenges to rural socio-economic development and national food security. According to the Chinese National Bureau of Statistics, the proportion of grain crops sowing area in China decreased from 70.48 to 69.71% between 2018 and 2021. The number of migrant workers increased by 2.52% (about 7.26 million), and the proportion of non-agricultural income of rural households increased from 63.34–65.31%^5^. In 2019, the rate of abandoned arable land in China was about 20%^3^, and the vacancy rate of rural housing was about 10%^6^. At the same time, the phenomenon of anti-urbanization is becoming more common, with urban residents moving to rural areas and migrant workers returning to their hometowns to start businesses. According to data from the Chinese Ministry of Agriculture and Rural Affairs, by the end of 2022, the number of people who returned to their hometowns to start businesses reached 12.2 million, driving the employment of over 50 million people in rural areas^7^. Therefore, studying these typical behaviors including non-agriculturalization, non-grainization, and anti-urbanization among rural households holds great significance for ensuring stable socio-economic development and food security.

Livelihood non-agriculturalization refers to the transforming process of rural households towards the industry and services for employment and relocating labour to urban areas. The main livelihood modes may include part-time farming^8,9^, semi-farmer and semi-labour^10^, and non-agriculture oriented^11^. Livelihood non-grainization refers to the adjustment of production structure within the agricultural sector by rural households, manifested as a decrease in the willingness to grow grain crops and an increase in the proportion of economic crops. Non-grain farmers are shifting from traditional subsistence farming to vocational^12^, scale-oriented^13^, and specialized farming^11,14,15^in pursuit of economic efficiency. Livelihood non-agriculturalization is mainly driven by the dual urban-rural system^16,17^, such as unequal public services and development opportunities^18–20^, urban-rural income disparity^21,22^, and the price scissors between industrial and agricultural products^23^. It is also influenced by micro factors related to farmers’ livelihood assets. For example, human asset and financial asset are seen as the key factors influencing non-agricultural livelihoods^10,24^while natural asset directly impacts on agricultural production^11^. As for livelihood non-grainization, the main driving force is that grain crops are less profitable compared to cash crops^15,25^. Industrial and commercial capital moving to rural areas^26^and farmland transfer^27^are also important factors driving non-grainization. Livelihood Anti-urbanization refers to these non-agriculture oriented households who settle and work in urban areas return to their hometowns for entrepreneurship or farming due to economic fluctuations, employment pressure, hometown sentimentality, and nostalgia for their homeland^28^. The anti-urbanization of non-agriculture oriented households is influenced by multiple factors such as employment benefits, living conditions, social integration, entrepreneurial environment, and livelihood assets^29^.

Livelihood behavior of non-agriculturalization, non-grainization, and anti-urbanization faces a series of risks, such as market fluctuations^30–33^, social policies^34,35^, environmental pollution^36–38^, and natural disasters^39–43^. The decision-making process regarding these behaviors is largely influenced by various risks and farmersʼ preferences. Existing studies on farmers’ risk preferences mainly focus on climate change adaptation^44^, adjustment of planting structures^45^, introduction of new technologies^46^, and large-scale farming^47^. Research indicates that the majority of Chinese farm households are risk-averse^44^, and they do not always follow the rational economic person assumption in decision-making^47^. Instead, they tend to exhibit risk aversion, aiming to minimize losses and prioritize safety, and are influenced by numerous factors such as social assets, education level, health status, age, etc^47,48^. Despite this, current research remains fragmented with scarce systematic analysis on rural risk preferences and livelihood decision-making, especially lacking analysis on the typical livelihood behaviors (non-agriculturalization, non-grainization, anti-urbanization) of typical rural households in typical regions.

Ruijin City, as a typical hilly and mountainous area in southern China, possesses good ecological environment and abundant natural resources. However, its complex terrain and inconvenient transportation, together with the fragmentation of land, result in the small-scale and inefficient agriculture^11^. During the past decades, Ruijin City has gone through rapid industrialization and urbanization, fostering wide-spread commercialization and globalization in agricultural sector and breeding diverse non-farm and pluriactive livelihoods in rural areas^11^. As the consequence, some farm households shifted their focus to developing specialty crops and animal husbandry, such as navel oranges, tobacco, lotus seeds, and poultry, while the others seeked non-agricultural employment opportunities outside their hometown. Livelihood transitions to non-agriculturalization, non-grainization, and even anti-urbanization are widespread in rural Ruijin City.

Therefore, this study takes Ruijin City, and 469 rural households from 30 natural villages, as the cases to explore farmers’ risk preferences and the livelihood transition mechanisms of typical rural households (vocational farmer household, semi-farmer and semi-labour household, non-agriculture oriented household) regarding non-agriculturalization, non-grainization and anti-urbanization. The study aims to answer two scientific questions: (1) What are the characteristics and influencing factors of risk preferences among rural households in the hilly and mountainous areas of southern China? (2) How do risk preferences, asset endowments, and other factors affect rural livelihood transitions toward non-grainization, non-agriculturalization, and anti-urbanization? This study may help local governments efficiently control the non-grainization and non-agricultural utilization of arable land resources, and rationally guide the capital influx and anti-urbanization in rural China. The research findings may provide references for worldwide rural revitalization and food security, especially in the developing country.

## Research framework and methodology

### Theoretical framework

**Fig. 1:**
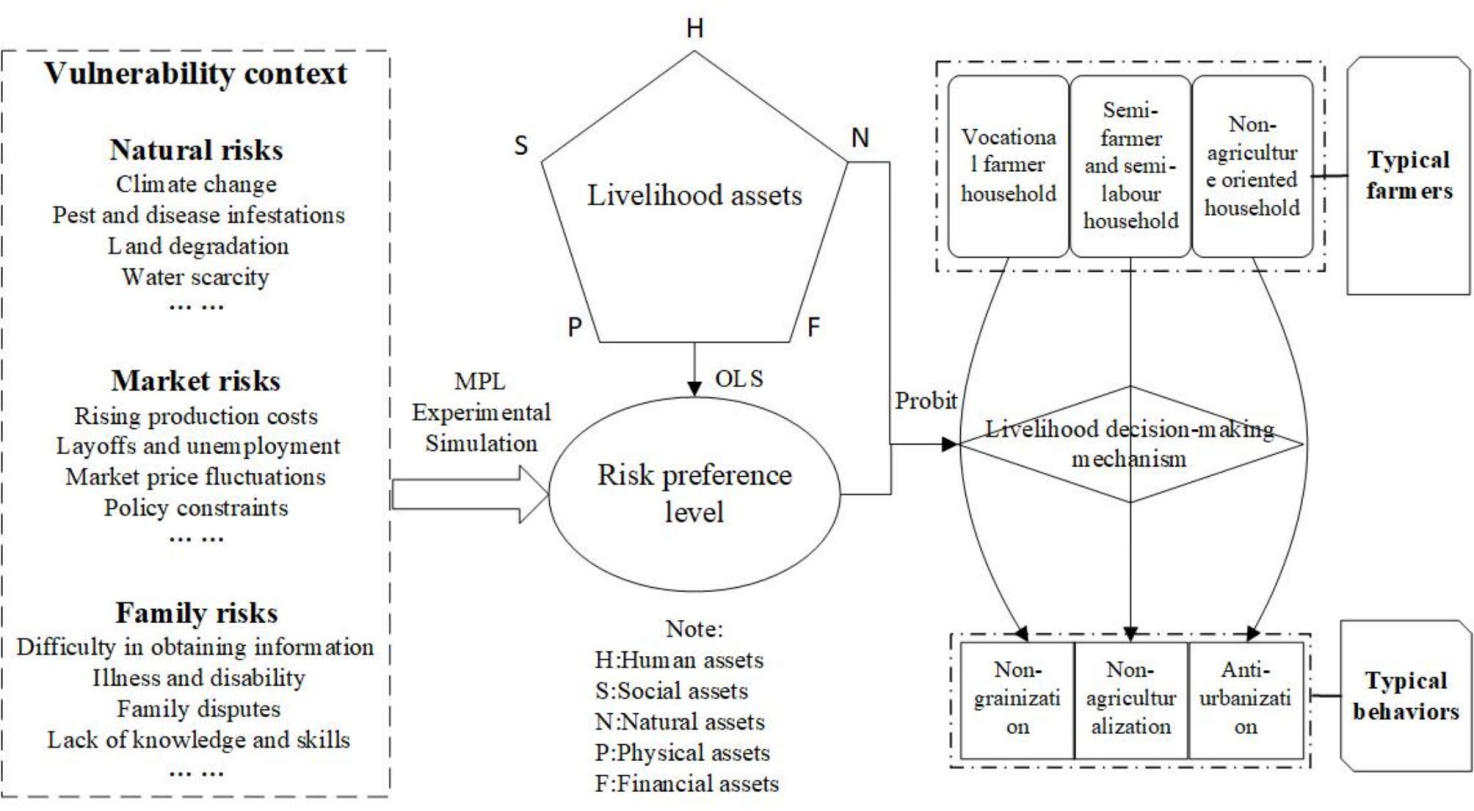
Theoretical Framework for Rural Livelihood Transition Analysis.

The SLA framework proposed by the Department for International Development^49^has multiple merits in livelihood analysis and possesses the highest degree of scholarly acceptance and empirical use. Composed of five parts —vulnerability context, livelihood capital, transformation of structures and institutions, livelihood strategy and livelihood outcomes—this framework treats household livelihoods as existing within certain vulnerability contexts, in which the capital pentagon is the core of the sustainable livelihoods framework^50^. In this study, we modified the SLA framework from the perspective of farmersʼ risk preference and incorporated multiple factors that may influence rural livelihood decision-making into the vulnerability context. As shown in Fig. 1, the livelihood transformation of rural households is a complex decision-making process, influenced by vulnerability context, livelihood assets, and risk preferences. Especially in situations where market information is limited and production and livelihood risks are uncontrollable, the livelihood transformation decision of rural households must consider not only maximizing benefits, but also minimizing risks. According to the sources of risk, rural households’ livelihood risks can be divided into natural risks, market risks, and family risks. Among them, natural risks mainly originate from the natural environment, such as climate change, geological disasters, pests and diseases, land degradation, etc.; market risks stem from macroeconomic cycle changes and market fluctuations, such as rising production costs, falling product prices, and unemployment. Family risks belong to internal risks of rural households, such as illness, disability, family disputes, lack of knowledge and skills, and inadequate information channels. These potential risks together constitute the vulnerability background of rural household livelihoods. Different households often exhibit different attitude preferences when dealing with risk, which can be divided into different levels such as risk preference, risk neutrality, and risk aversion. This difference in attitude preferences may be rooted in the differences in household asset endowments, including natural assets, human assets, physical assets, financial assets, and social assets. Therefore, there are complex interactions among livelihood risks, livelihood assets, risk preferences, and the livelihood transformation decisions of rural households. The underlying mechanisms require further study.

Based on extensive household visits and questionnaire surveys, this paper classifies rural households into traditional small-scale farmers, vocational farmer households, semi-farmer and semi-labour households, non-agriculture oriented households, and retired farmer households, according to factors such as the proportion of annual net income, occupation, work location, and education level (see Table 1). Among them, traditional small-scale farmers have disappeared under the drive of urbanization and industrialization, while retired farmers mainly rely on their children’s support and subsidies without livelihood capability. Hence, we do not discuss these two types of rural households in detail. Instead, we give more focus to the typical livelihood behaviors of these three kinds of typical households: non-grainization among vocational farmer households, non-agriculturalization among semi-farmer and semi-labour households, and anti-urbanization among non-agriculture oriented households. Vocational farmer households are those with agricultural income accounting for 70% or more of their total income (in Ruijin City, vocational farmer households are mainly professional growers of characteristic crops such as navel oranges, lotus seeds, and tobacco). They are modern agricultural practitioners who take agriculture as their vocation. They can proficiently conduct agricultural production and management, possess certain innovation and investment awareness, and are willing to try diversified inputs and new agricultural production technologies. Households of this type are mainly driven by economic interests. Most of them are willing to or have already planted economic crops with higher added value The phenomenon of non-grainization is very prominent. Semi-farmer and semi-labour households are households whose total agricultural and non-agricultural income accounts for 70% or more, but each account for less than 70%. As a transitional livelihood type between agriculture and non-agriculture, they balance agricultural production and non-agricultural employment. Under the drive of urbanization and industrialization, this type of household tends to further transform into non-agricultural sectors. However, due to the instability of non-agricultural employment and high risks in agricultural production, their non-agricultural behavior has uncertainties. Non-agriculture oriented households mainly engage in non-agricultural employment, with the standard of non-agricultural income accounting for 70% or more, including non-agricultural workers engaged in low-end service industries and manufacturing industries, and non-agricultural technicians with higher education levels and stable employment. Due to the risks of macroeconomic fluctuations, employment pressure, and social changes, non-agriculture oriented households may have the possibility of returning to their hometowns for entrepreneurship or farming. At the same time, some rural elites show anti-urbanization behavior due to their affection for their hometowns, for example, some returned to the countryside to operate leisure and sightseeing agriculture and health-preserving agriculture.

Therefore, this paper focuses on analyzing the typical livelihood behaviors (non-grainization, non-agriculturalization, and anti-urbanization) and decision-making mechanisms of typical households (vocational farmer households, semi-farmer and semi-labour households, and non-agriculture oriented households). First, the influencing factors of risk preference of rural households in the southern hilly and mountainous areas are identified based on an OLS regression given the function of livelihood assets on risk preference. Then, using livelihood assets and risk preference variables, a Probit regression is applied to analyze the decision-making mechanisms of typical households, considering non-grainization, non-agriculturalization, and anti-urbanization as typical livelihood behaviors.

**Table 1:** Livelihood pattern in rural Ruijin City.

Livelihood modes	Traditional small-scale farmer household	Vocational farmer household	Semi-farmer and semi-labour household	Non-agriculture oriented household	Retired farmer household
Income Composition	Totally subsistence	Agricultural income accounts for 70% or more.	The total proportion of agricultural income and non-agricultural income is greater than 70%, with each individual proportion being less than 70%	Non-agricultural income accounts for 70% or more	Subsidy income accounts^a^ for 70% or more.
Livelihood characteristics	Mainly engaged in meticulous farming and small-scale management to meet self- consumption or a small amount of cash needs.	Possess certain professional skills and a strong market awareness, proficient in agricultural production and management, with the main source of income coming from agriculture.	Balancing agriculture and non-agriculture, engaging in farming at home during busy farming seasons, and seeking off-farm employment during idle farming periods, maintaining a balance between agricultural and non-agricultural income proportions.	Mainly engaged in non-agricultural employment such as the service industry, manufacturing industry, construction industry, etc., with relatively high household income and consumption levels.	Elderly households without labour capacity who rely on children’s support and various forms of subsidy income
Future trend	Disappeared	Spring up	Transitional	Increasing	Existing chronically
Sample	0	80	65	266	58

^a^ Subsidized income mainly refers to subsidies from cropland retirement, pension subsidies, ecological compensation, child support and rural minimal social security subsidies.

### Study area

Ruijin City, amid the hilly and mountainous region of southern China, has experienced long-term urbanization and significant rural transformation. The case of Ruijin City has typical characteristics and specificities in the aspect of geographical environment and social economy, which can be seen as the epitome of rural transitions in China and even other worldwide areas. (1) In terms of geographical environment, Ruijin City possesses complex topography, excellent environment yet limited cropland. Ruijin City lies in the junction of Jiangxi Province, Fujian Province and Guangdong Province, E115°42′～116°22′, N25°30′～26°20′,and lies at the western foot of the southern Wuyi Mountains, the upper reaches of the Gongshui River, and the eastern source of the Ganjiang River. Ruijin has a subtropical monsoon humid climate, abundant rain and heat resources, complex river system, and an annual average rainfall of 1,710 mm. The total land area is 2,448 km^2^with high terrain surrounding the central lowlands, which creates complex topography. As a result, Ruijin city’s ecosystems and natural resources are rich and diverse, including forests (1,850 km^2^), croplands (238 km^2^), garden lands (40.33 km^2^), water reserves (1.98 billion m^3^), etc. (Fig. 3), playing important roles in local residents’ livelihoods and socioeconomic development. (2) In terms of economic characteristic, Ruijin City shares many similarities with other rural areas around the world. Before the industrialization and urbanization launched in 1978, Ruijin City was a typical agricultural area mainly engaged in the primary industry of plantation (rice) and livestock husbandry (pig). After 1978, agriculture in this area turned to commercialization and globalization, and produced more cash crops and husbandry products based on the mountainous resources and environment, for example, navel orange, lotus, tobacco, beef. In 2022, Ruijin has a permanent population of 613,894 and an urbanization rate of 65.20%. The per capita GDP was 29,468 yuan (RMB), and the per capita disposable income of rural residents was 16,573 Yuan (RMB). (3) In terms of social change, the rapid urbanization and industrialization has also fostered more diverse villages and *pluriactive* livelihoods. Due to the mountainous location, inconvenient transportation, frequent natutal disasters, and the rising labour cost, farmers seeked non-agricultural employment. The abandonment of cultivated land and the problems of non-agricultural and non-grain utilization of farmland have become prominent issues. In the context of national food security, Ruijin City must curb the “non-agriculturalization” and the “non-grain utilization” of farmland. Therefore, it is typical and representative to study the livelihood transitions towards non-agriculturalization, non-grainization, and anti-urbanization in Ruijin City.

**Fig. 2:**
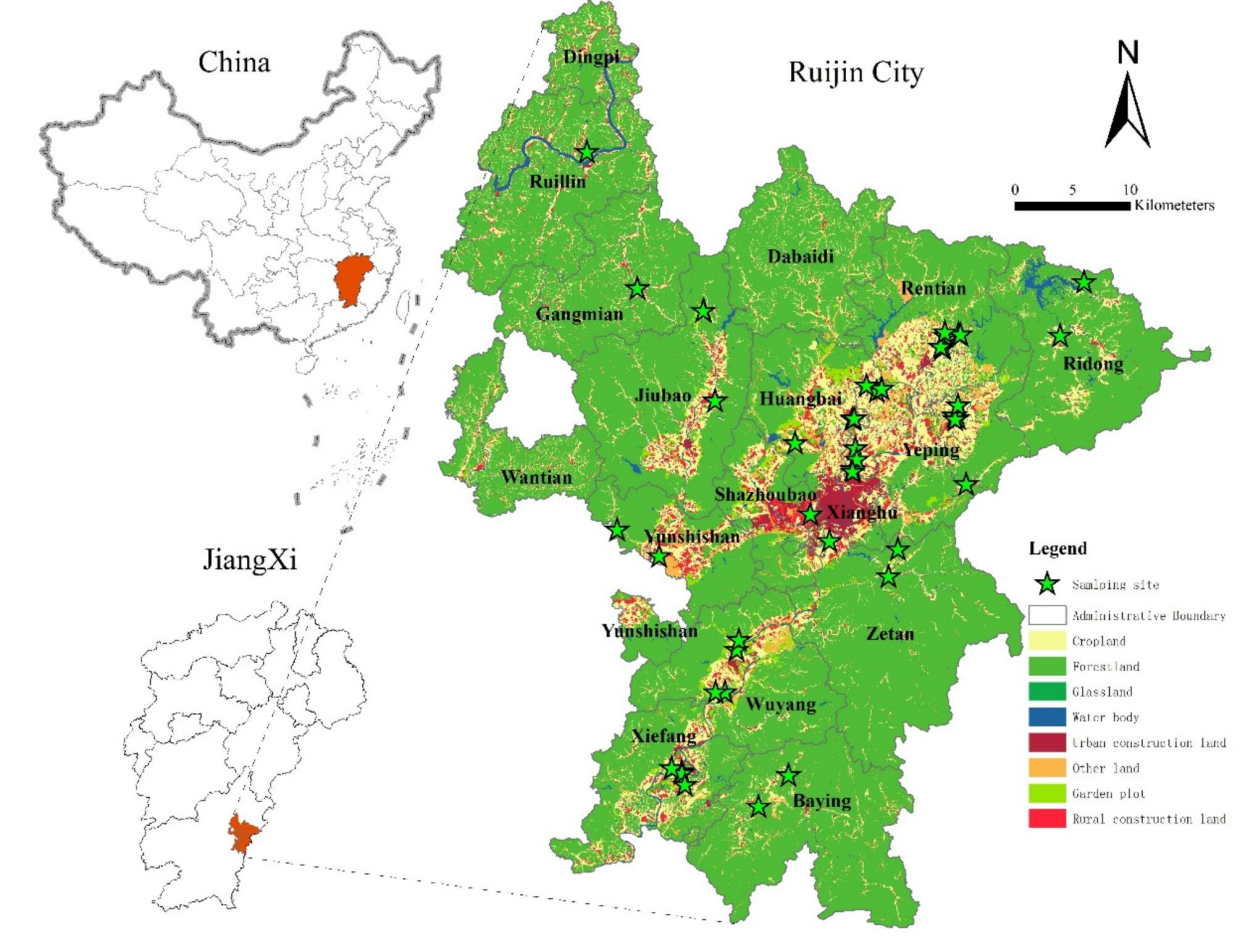
Location of the study area.

### Data collection

In January 2020, we conducted a field investigation and household surveys on 30 natural villages in 13 townships of Ruijin City through Participatory Rural Appraisal (PRA) and stratified random sampling methods (Fig. 2). A total of 479 questionnaires were distributed, and 469 valid questionnaires were collected. The effective rate of the survey was 97.9%. It included 80 vocational farmer households, 266 non-agriculture oriented households, 65 semi-farmer and semi-labour households, and 58 retired farmers. The questionnaire mainly covered information on demography, livelihood assets, risk preferences, and livelihood selection behavior. During the questionnaire survey, the research team identified household categories based on the household’s occupation and main source of income. The research team surveyed the main livelihood risks through open-ended questions, and measured household risk preferences using a multi-price list experiment. Three core questions were designed with a dichotomous option to inquire about the livelihood adjustment strategies of vocational farmer households, semi-farmer and semi-labour households, and non-agriculture oriented households, namely, “Once risk happens, will you plant non-grain economic crops?”, “Once risk happens, will you choose non-agricultural transformation?”, “Once risk happens, will you return to your hometown for entrepreneurship or farming?”.

### Methodology

#### Livelihood asset indicator system

According to the sustainable livelihood framework, we constructed an indicator system for livelihood asset assessment (Table 2). Among these, the family labour force and education level of the household leader were selected as the indicators of human assets. As the fundamental natural asset for households, the land management area was chosen as the indicator of natural asset variables. Rural physical assets mainly include living assets and production assets. Therefore, housing conditions and the quantity of production machines were selected as the indicators of physical asset variables. Financial assets mainly refer to the funds and credit conditions available to households; thus, this study selected annual net income and credit conditions as indicators of financial asset variables. Social assets for households primarily consist of their social networks and access to public facilities; hence, this study selected the intimacy of relatives and friends and the distance to public facilities as indicators of social asset variables.

**Table 2:** Livelihood asset indicator system.

Livelihood assets	Index	Value assignment	Mean Value	Standard deviation
Human assets	Family labour force	Sum each item according to the age assignment of members in a household. Assign 0 for the disabled, the children younger than 5, and the seniors older than 70; assign 0.2 for the children between 6 and 10; assign 0.6 for the teenagers between 11 and 17; assign 1 for the adults between 18 and 60; assign 0.5 for seniors between 61 and 70	2.591	1.086
	Education level of household leader	Assign 1 for the illiteracy; assign 2 for the primary school; assign 3 for the middle school; assign 4 for the high school and technical secondary school; assign 5 for the junior college and above	2.409	0.840
Natural assets	Land management area	The total area of cultivated land, garden land and woodland actually managed by the family/mu	4.367	16.114
Physical assets	Housing assets	= Decoration cost/ Wan Yuan × 0.7 + Housing Area/square meter × 0.3	40.423	15.726
	Production assets	Sum each item according to the number of production tools	0.608	4.000
Financial assets	Annual net income	According to the research data, present it as annual net household income/ Yuan	7.605	15.858
	Credit condition	Set a dichotomous variable, assign 1 for possess otherwise 0 and sum each item	0.304	0.461
Social assets	Intimacy of relatives and friends	According to the research data, represented as annual household cash gifts	0.365	0.366
	Condition of public facilities	Add up the distances to the nearest primary school, clinic and bus stop	10.520	16.495

Referring to existing research, rural livelihood assets are calculated based on the compound weight method. The main steps are as follows: (1) Data standardization. Because the units of the indicators are different, it is necessary to standardize each indicator. This study uses the Min-Max normalization method to convert the values of indicators of different categories ranging from 0 to 1. (2) Determine weights. This study adopts the entropy method to determine the weights of indicators, which uses information entropy to calculate the weights of each indicator. (3) Calculate the asset abundance. Based on the standardized results and weights of each indicator, the numerical value of household asset abundance is determined. The calculation formula is as follows:

1 $$\:\begin{array}{c}S=0.2\sum\:_{\text{i}=1}^{5}{\text{T}}_{\text{i}}=0.2\sum\:_{\text{i}=1}^{5}\sum\:_{\text{j}=1}^{1}{\text{W}}_{\text{i}\text{j}}{\text{X}}_{\text{i}\text{j}}\end{array}$$

In the formula, $\:{\text{T}}_{\text{i}}$ represents the measure of asset abundance for each category, $\:{\text{W}}_{\text{i}\text{j}}$ represents the weight of the j indicator for the i category of livelihood assets, $\:{\text{X}}_{\text{i}\text{j}}$ represents the standardized result of the j indicator for the i category of livelihood assets, and S represents the total measure of asset abundance.

#### Multiple price list (MPL)

The multiple price list (MPL) experiment was developed based on Brick et al.^51^, which is a variation of the widely used Holt-Laury-type measure^52^. The MPL is a standard format, whereby subjects are provided with a fixed array of paired lottery options and choose one option per pair. This experimental approach can be designed to measure participants’ decision-making behaviors and risk preferences when faced with various risk and reward scenarios. It exhibits several key characteristics: ease of implementation, comprehensibility, rational design, and the capability to account for relative risk preferences. Thus, it can promote honest answers^53^.

As illustrated in Table 3, there are two job options, A and B. Job A offers a stable income, whereas Job B carries a risk, with a 50% chance of earning $20,000 per year and a 50% chance of no income. Under eight different scenarios where Job A’s stable income varies from $2,000 to $20,000, participants are required to respond to each scenario.

In the first scenario, choosing Job A guarantees a 100% chance of earning $20,000, while choosing Job B presents a 50% chance of earning $20,000 and a 50% chance of no income. This initial question is unrelated to risk preference; it merely tests whether participants understand the instructions. Across the eight scenarios, the expected income from choosing Job A gradually decreases, while the expected income from choosing Job B remains constant. Assuming constant relative risk preferences, participants’ risk preferences are directly correlated with their switch from option A to option B. For instance, a very risk loving individual might choose B in the second scenario, whereas a highly risk averse individual would consistently choose A across all eight scenarios. From an expected income perspective, the fourth scenario presents an equal expected income, suggesting that a risk neutral individual would opt for A in the first three scenarios and B in the subsequent ones.

Step One involves the investigator introducing the experimental rules to the participants, familiarizing them with the game rules, and presenting a list as shown in Table 3, but only revealing the content of the first three columns. Participants must choose for each scenario.

Step Two occurs after the participants have made their choices across all eight scenarios. One of these scenarios is randomly selected, and the participant’s choice in this scenario determines their reward.

Step Three involves determining the participant’s scenario number and rewarding them based on their choice. If they chose option A, the reward is based on a thousandth of the amount specified for that option. If they choose option B, a coin toss determines the reward. For example, if a participant’s randomly selected scenario is the sixth, and they chose option A, they receive $6; if they chose option B, a coin toss decides whether they receive $20 or no reward at all.

**Table 3:** Experimental design on farmers’ risk preferences.

Livelihood Projects	Option A Income	Option B Income	Degree of Risk Preference
1	20,000	20,000 or 0	-
2	15,000	20,000 or 0	Very risk loving
3	12,000	20,000 or 0	Risk loving
4	10,000	20,000 or 0	Risk neutral
5	8,000	20,000 or 0	Slightly risk averse
6	6,000	20,000 or 0	Risk averse
7	4,000	20,000 or 0	Very risk averse
8	2,000	20,000 or 0	Highly risk averse

-This line of farmers who chose Project B in the experiment misunderstood it, as Project A was certain to provide returns equal to or higher than Project B.

#### Ordinary least squares method

Linear regression is a widely used statistical analysis method. The relationship equation for Ordinary Least Squares (OLS) is as follows:

2 $$\:\begin{array}{c}Y={{\upbeta\:}}_{0}+{{\upbeta\:}}_{1}{\text{x}}_{1}+{{\upbeta\:}}_{2}{\text{x}}_{2}+\cdot\:\cdot\:\cdot\:+{{\upbeta\:}}_{\text{k}}{\text{x}}_{\text{k}}+\epsilon\end{array}$$

In Eq. (1), y represents risk preference, $\:{{\upbeta\:}}_{0}$ denotes the constant term, $\:{\text{x}}_{\text{k}}$ represents the endowment variables of farmers, including family labour force, education level of household leader, land management area, housing assets, etc. $\:{{\upbeta\:}}_{\text{k}}$ are the corresponding coefficients, and $\epsilon$ is the random error term. The basic idea is to find the optimal parameters of the model by minimizing the sum of squared residuals.

#### Probit regression

Probit regression is a generalized linear model based on the normal distribution, used to solve binary classification problems. The purpose of Probit regression is to predict the probability of an observation belonging to a certain category by the linear combination of independent variables. In Probit regression, we assume that the dependent variable follows a binomial distribution, i.e.:

3 $${y}_i\:\sim\:Bin({n}_{i},{p}_{i})$$

Where $\:{\text{y}}_{\text{i}}$ represents the outcome of the i observation, $\:{\text{n}}_{\text{i}}$ represents the number of trials, and $\:{\text{p}}_{\text{i}}$ represents the probability of success. Since Probit regression is based on the normal distribution, we transform $\:{\text{p}}_{\text{i}}$ as follows:

4 $$\:\begin{array}{c}\Phi\:\left({z}_{i}\right)={p}_{i}\end{array}$$

Where $\:{\Phi\:}\left({z}_{i}\right)$ is the standard normal cumulative distribution function, and $\:{z}_{i}$ is a linear combination expressed as:

5 $$\:\begin{array}{c}{z}_{i}={\beta\:}_{0}+{\beta\:}_{1}{x}_{i1}+{\beta\:}_{2}{x}_{i2}+\dots\:+{\beta\:}_{p}{x}_{ip}\end{array}$$

Where $\:{\beta\:}_{0},{\beta\:}_{1},{\beta\:}_{2}$, and so on are the regression coefficients, $\:{x}_{i1},{x}_{i2},{x}_{i2}$, and so on are the independent variables of the i observation. The template of Probit regression is to estimate the regression coefficients $\:\beta\:$ by maximizing the log-likelihood function L. The specific steps are as follows: (1) Select the independent variables and build the Probit regression model. (2) Utilize numerical optimization algorithms to estimate the regression coefficients $\:\beta\:$ by maximizing the log-likelihood function L. (3) Based on the estimated coefficients, predict whether new samples belong to a certain category and calculate the probability of belonging to that category.

To analyze the impacts of livelihood assets and risk preferences on non-agriculture and non-grain, this study employs the Probit regression model, expressed as follows:

6 $$\:\begin{array}{c}{\text{Y}}_{\text{i}}=\alpha\:+\sum\:{{\upalpha\:}}_{\text{i}}{\text{X}}_{\text{i}}+{\text{p}}_{1}{\text{D}}_{\text{i}}+\epsilon\end{array}$$

Where ($\:{\text{Y}}_{\text{i}}$) is a binary dummy variable (representing the non-grain behavior of vocational farmer households, the non-agriculture behavior of semi-farmer and semi-labour households, and the anti-urbanization behavior of non-agriculture oriented households, with 1 = Yes and 0 = Others). $\:{\upalpha\:}$ is the intercept, $\:{{\upalpha\:}}_{\text{i}}$ are the corresponding coefficients, $\:{\text{X}}_{\text{i}}$ represents household asset endowment variables including family labour force, education level of household leader, land management area, housing assets, etc., and $\:{\text{D}}_{\text{i}}$ denotes the level of household risk preference. $\epsilon$ is the random error term.

## Results

### Characteristics of rural livelihoods in the hilly and mountainous area

**Fig. 3:**
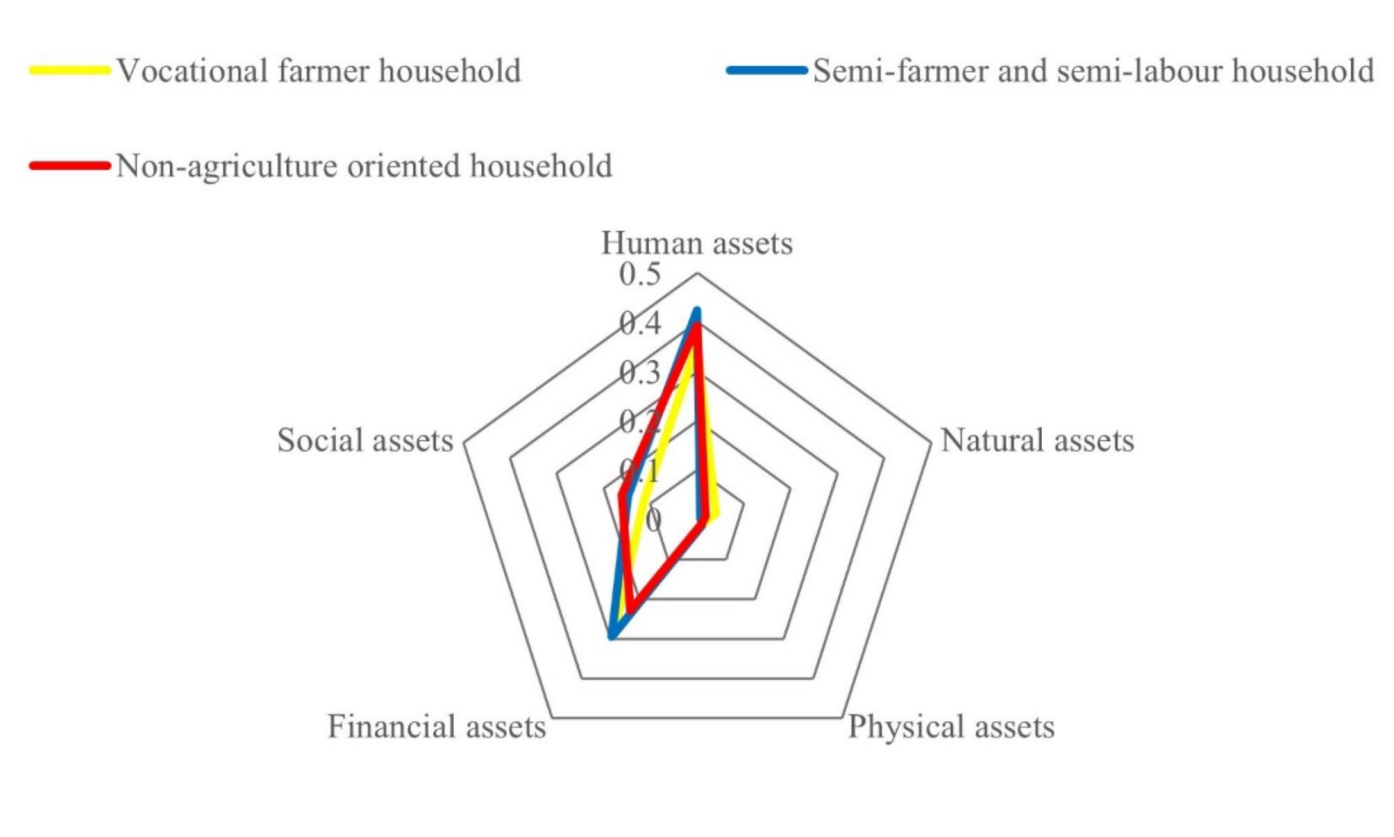
Livelihood assets of different rural households in Ruijin City.

**Fig. 4:**
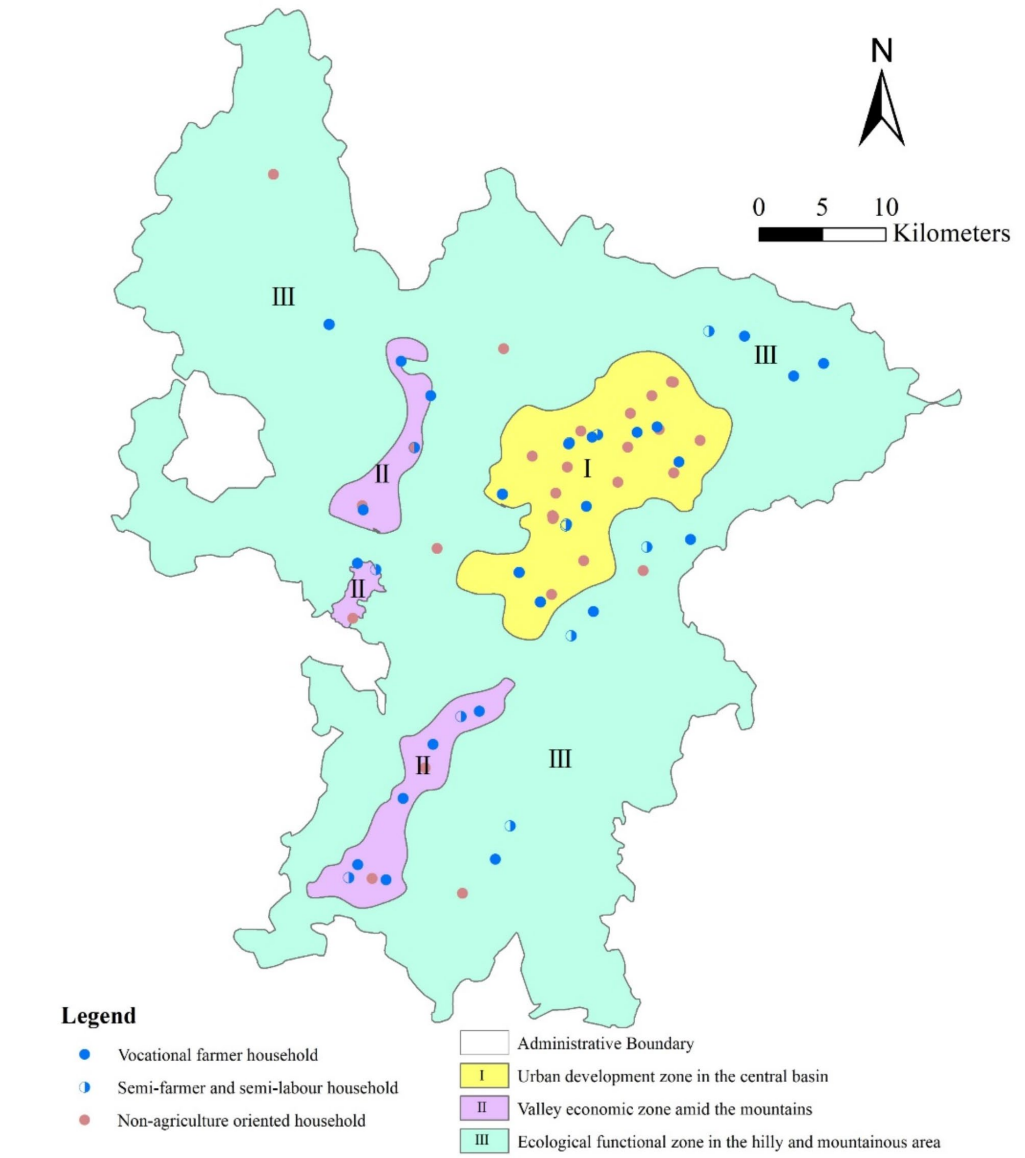
Spatial pattern of rural livelihoods in Ruijin City.

**Fig. 5:**
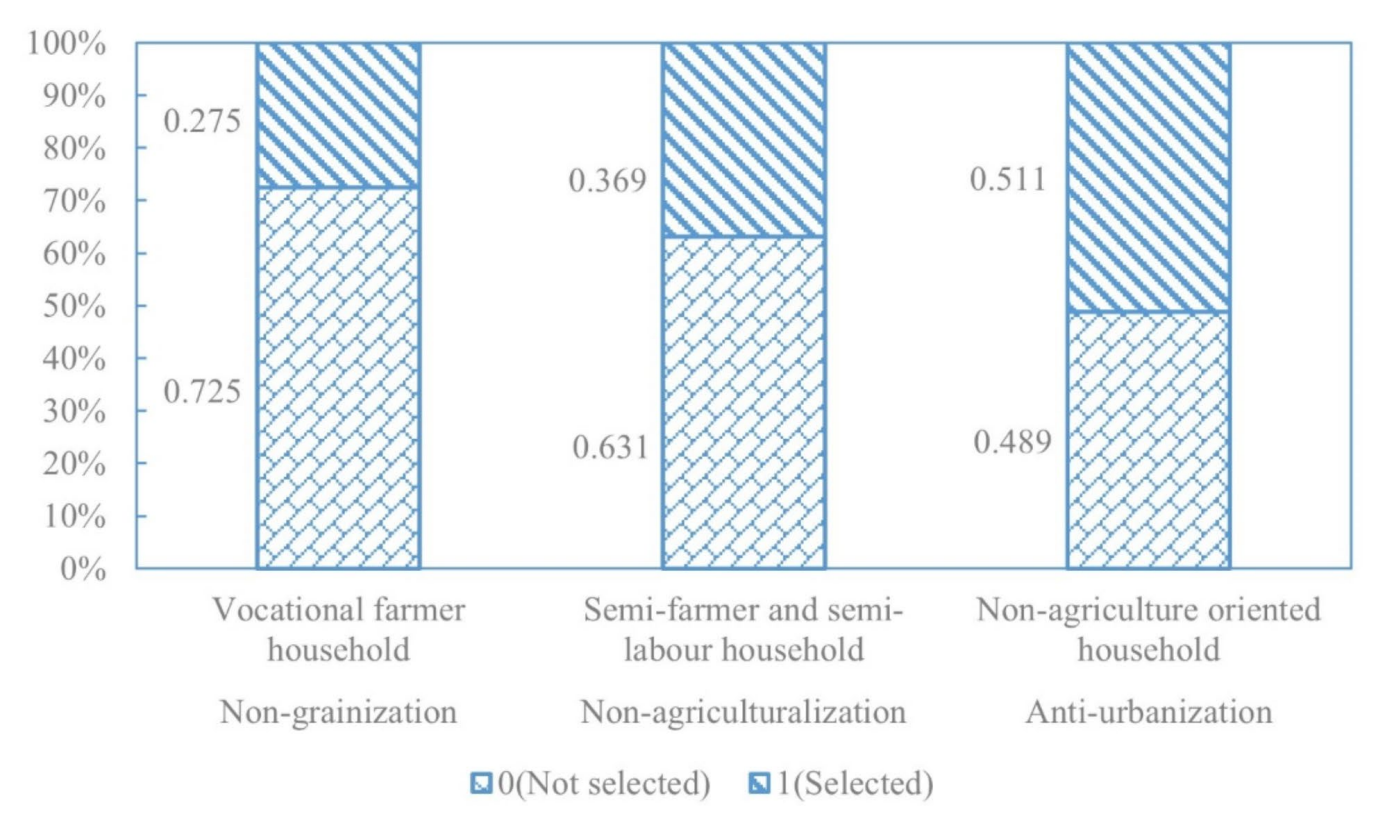
Livelihood choice of rural household in Ruijin City.

As depicted in Table 1, there are three main categories of rural livelihood in hilly and mountainous areas: vocational farmer households, semi-farmer and semi-labour households, and non-agriculture oriented households. According to the estimation, the abundance of livelihood assets among these three typical rural households ranks as follows: Non-agriculture oriented households (0.159) > Semi-farmer and semi-labour households (0.149) > Vocational farmer households (0.143). Among them, non-agriculture oriented households have the highest total assets, and their human assets (0.424), physical assets (0.016), and financial assets (0.216) all rank first (see Fig. 3). It indicates that non-agriculture oriented households have an abundant labour force, higher education levels, and the highest production and living standards. This kind of household is mainly distributed in the urban development zone in the central basin and the ecological functional zone in the hilly and mountainous area in Ruijin City (see Fig. 4). For non-agriculture oriented households residing in the central basin urban development zone, the infrastructure for transportation and education is well-established with smooth information flow and more job opportunities. Most of them possess stable employment and good welfare benefits. On the other hand, non-agriculture oriented households in the ecological functional zone in the hilly and mountainous area are forced to leave their hometown for non-agricultural work due to livelihood pressures. However, the empolyment is always temporary and highly unstable. Risks for non-agriculture oriented household are mainly economic fluctuations, unemployment, aging, and illness. When facing risks, 51.1% of non-agriculture oriented households choose to return to their hometowns for entrepreneurship or farming (see Fig. 5).

**Fig. 6:**
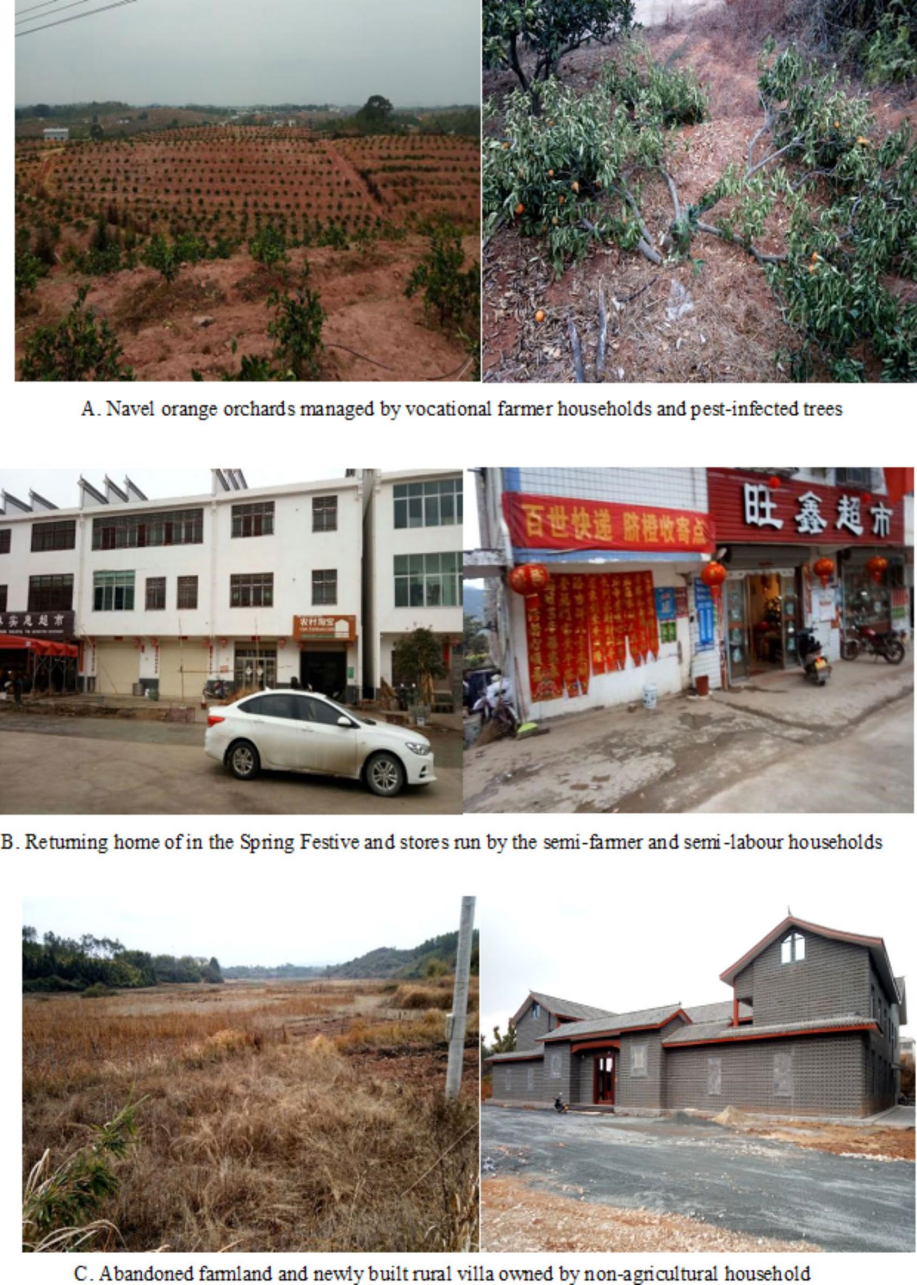
Livelihood behavior of typical rural households in the hilly and mountainous area.

The total amount of livelihood assets of vocational farmer households is the lowest, with natural assets (0.040) ranking first, while human assets (0.353) and social assets (0.096) both rank the lowest. Vocational farmer households have a large amount of land resources for large-scale agricultural production, but they relatively lack labour force and have weak social relationship networks. It is consistent with the characteristics of specialized farmers in hilly areas. This kind of farmer is mainly distributed around towns and in the special economic zones of mountainous valleys. Vocational farmer households in the central basin urban development zone are closer to the market. Besides growing grains, they often plant seasonal vegetables, fresh fruits, and other high-value, perishable economic crops. Farmers in the special economic zones of mountainous valleys rely on the excellent ecological environment and abundant arable land and garden resources to grow special economic crops, such as navel oranges (Fig. 6), tobacco, and lotus seeds. Their main livelihood risks include pests and diseases, market fluctuations, and climate change. When facing risks, 27.5% of vocational farmer households choose to continue non-grain cultivation of economic crops.

The total amount of livelihood assets of semi-farmer and semi-labour households ranks second, with social assets (0.150) ranking first, human assets (0.394) and natural assets (0.018) being moderate, but financial assets (0.169) and physical assets (0.013) both ranking last. It indicates that this kind of rural household possesses wide social relationships, and multiple job information channels, but low income and living standards. As a form of diversified livelihood, semi-farmer and semi-labour households exist widely in the special economic zones of mountainous valleys and the ecological functional areas. Women and the elderly engage in household farming, while male labourers work outside when farming is idle yet return to farming during busy farming periods. Diversifying into semi-farming and semi-labour activities can not only broaden income sources but also effectively mitigate risks. Therefore, when facing risks of non-agricultural unemployment and agricultural disasters, only 36.9% of semi-farmer and semi-labour households choose to transform entirely to the non-agricultural sector.

### Risk preferences of farmers in the hilly and mountainous area

As shown in Table 4, the mean value of risk preference of the overall sample is 1.48, with 76.86% being risk averse and 60.67% being highly risk averse. Only 12.85% are risk loving, and 10.28% are risk neutral. Specifically, vocational farmer households have the highest risk preference value (1.75), among which the risk averse accounting for 69.62%, the risk neutral and the risk loving each accounting for 15.19%. Non-agriculture oriented household have the lowest risk preference level (1.30), with 78.95% being risk averse, 12.55% being risk loving, and 8.50% being risk neutral. Semi-farmer and semi-labour households have a moderate risk preference value (1.44), with 77.78% being risk averse, and 11.11% each for risk neutral and risk loving.

**Table 4:** Risk preferences of different farmers.

No. of times subject choose risky option	Switch point to the risky option	Degree of risk preference	Total sample		Vocational farmer households		Semi-farmer and semi-labour household		Non-agriculture oriented household	
			No. of subjects	%	No. of subjects	%	No. of subjects	%	No. of subjects	%
0	Always option A	Highly risk averse	236	60.67%	46	58.23%	40	63.49%	150	60.73%
1	2	Very risk averse	22	5.66%	5	6.33%	3	4.76%	14	5.67%
2	3	Risk averse	27	6.94%	2	2.53%	5	7.94%	20	8.10%
3	4	Slightly risk averse	14	3.60%	2	2.53%	1	1.59%	11	4.45%
4	5	Risk neutral	40	10.28%	12	15.19%	7	11.11%	21	8.50%
5	6	Risk loving	19	4.88%	3	3.80%	4	6.35%	12	4.86%
6	7	Very risk loving	15	3.86%	3	3.80%	3	4.76%	9	3.64%
7	Always option B	-	16	4.11%	6	7.59%	0	0.00%	10	4.05%

According to Table 5, farmers’ risk preferences are closely related to the family labour force, education level of household leader, housing assets, credit condition, the intimacy of relatives and friends, and access to public facilities. Specifically, vocational farmer households’ risk preferences are significantly negatively correlated with access to public facilities. This is because the agricultural products produced by vocational farmer households, especially perishable fruits and vegetables, are sensitive to transportation distance, and farmers have weaker risk-bearing capacity. Non-agriculture oriented households’ risk preferences are significantly positively correlated with the family labour force, education level of household leader, credit condition, and intimacy of relatives and friends. For each unit increase in the family labour force, education level of household leader, credit condition, and intimacy of relatives and friends, the non-agriculture oriented households’ risk preference levels will increase by 0.244, 0.194, 0.185, and 0.166 units, respectively. This is consistent with the characteristics of non-agriculture employment requiring higher labour quality and the social network of farmers. The risk preferences of semi-farmer and semi-labour households are significantly positively correlated with the education level of household leader and housing assets. For each unit increase in the education level of household leader and housing assets, the risk preference level of semi-farmer and semi-labour households will increase by 0.220 and 0.225 units respectively. This is because a higher education level and better urban-rural housing imply a clearer understanding of risk and higher life security, which can help mitigate the impact of risk.

**Table 5:** OLS regression results of risk preference of typical household.

Livelihood Assets	Risk preference of overall sample		Risk preference of vocational farmer household		Risk preference of non-agriculture oriented household		Risk preference of semi-farmer and semi-labour household	
	COEF	SE	COEF	SE	COEF	SE	COEF	SE
Family labour force	0.148***	-0.054	0.016	-0.110	0.244***	-0.075	0.070	-0.134
Education level of household leader	0.166***	-0.049	0.063	-0.127	0.194***	-0.063	0.245**	-0.102
Land management area	-0.048	-0.049	-0.082	-0.059	-0.143	-0.294	-0.093	-0.332
Housing assets	0.113**	-0.050	0.143	-0.126	0.034	-0.070	0.209**	-0.085
Production assets	-0.027	-0.047	0.326	-0.461	-0.040	-0.049	0.567	-0.540
Annual net income	-0.056	-0.055	0.098	-0.110	-0.135*	-0.079	-0.002	-0.118
Credit condition	0.154***	-0.048	0.045	-0.114	0.185***	-0.061	0.095	-0.117
Intimacy of relatives and friends	0.090*	-0.049	0.037	-0.114	0.166**	-0.064	-0.048	-0.108
Condition of public facilities	-0.070	-0.049	-0.522*	-0.268	-0.042	-0.059	-0.084	-0.107
Constant	0	-0.047	-0.012	-0.142	-0.037	-0.079	-0.069	-0.110
Number	411		80		266		65	
R-squared	0.109		0.123		0.135		0.282	
F-statistic	5.426		1.093		4.441		2.405	
Adjusted R-squared	0.0886		0.0104		0.105		0.165	

***p < 0.01, **p < 0.05, *p < 0.1.

### Decision mechanism of livelihood transition of typical farmers

As shown in Table 6, the transition of vocational farmer households to non-grain cultivation is significantly and positively correlated with the family labour force, land management area, and housing assets, but negatively correlated with risk preference. Specifically, for each increase in the level of risk preference, the probability of vocational farmer households choosing non-grain cultivation will decrease by 0.251 times. This is because the production, processing, and sales of local economic crops such as navel oranges, tobacco, and lotus seeds have formed mature and stable industrial chains. These crops are mostly planted in mountainous and forested areas (navel oranges) or small cultivated land (tobacco, lotus seeds), conforming to the characteristics of small-scale farming in regions with more mountains and less land. Compared to grain cultivation, local non-grain cultivation offers higher economic benefits and lower opportunity costs and risks. In addition, for each unit increase in the family labour force, land management area, and housing assets, the probability of vocational farmer households choosing non-grainization will increase by 0.094, 0.162, and 0.187 times, respectively. This is because vocational farmer households with sufficient labour force, large land management area, and good housing conditions have the ability to expand the cultivation ratio and types of economic crops, thereby effectively enhancing the economic risk resistance of vocational farmer households.

The behavior of non-agriculture oriented households to revert to rural areas is significantly and positively correlated with annual net income, yet negatively correlated with risk preference and intimacy of relatives and friends. For each unit increase in annual net income, the probability of anti-urbanization by non-agriculture oriented households will increase by 0.134 times. Meanwhile, for each unit increase in risk preference level and intimacy of relatives and friends, the probability of anti-urbanization will decrease by 0.105 times and 0.065 times, respectively. This is because non-agriculture oriented households with higher income levels have a stronger desire to return to rural areas for health and leisure agriculture, but the high cost of social interaction has become a heavy burden on rural society and an important concern for non-agriculture oriented households returning to rural areas. From the risk perspective, returning to rural areas for farming is still considered a secure livelihood option by the majority of non-agriculture oriented households.

The transition to the non-agricultural sector by semi-farmer and semi-labour households is significantly and negatively correlated with land management area, yet positively correlated with annual net income. When other variables remain unchanged, for each unit decrease in land management area or each unit increase in annual net income, the probability of semi-farmer and semi-labour households transitioning to non-agriculturalization activities will increase by 0.438 times and 0.148 times, respectively. This is because farmers with larger land management areas have a stronger attachment to their land or possess certain agricultural skills and are unwilling to abandon traditional agriculture, while farmers with higher annual net incomes have a stronger desire to move to urban areas. Risk preference does not significantly impact the transition of semi-farmer and semi-labour households to non-agricultural activities, because these households face risks associated with both agricultural production and non-agricultural unemployment. Diversification remains the most effective strategy for risk mitigation.

**Table 6:** Probit regression of livelihood transition decision among typical farmers.

Variable	Non-grainization of vocational farmer household		Anti-urbanization of non-agriculture oriented household		Non-agriculturalization of semi-farmer and semi-labour household	
	COEF	SE	COEF	SE	COEF	SE
Family labour force	0.317*	0.094	-0.156	-0.062	-0.101	-0.037
Education level of household leader	0.324	0.096	-0.042	-0.017	-0.230	-0.083
Land management area	0.549*	0.162	-0.295	-0.118	-1.211*	-0.438
Housing assets	0.633**	0.187	-0.005	-0.002	0.240	0.087
Production assets	-0.017	-0.005	-0.057	-0.023	-0.928	-0.336
Annual net income	-0.092	-0.027	0.337***	0.134	0.408**	0.148
Credit condition	0.037	0.011	0.126	0.050	0.315	0.114
Intimacy of relatives and friends	-0.044	-0.013	-0.163*	-0.065	0.065	0.024
Condition of public facilities	-0.489	-0.144	-0.132	-0.053	-0.304	-0.110
Risk preferences	-0.850***	-0.251	-0.264***	-0.105	0.113	0.041
Constant	-0.968***		-0.021		-0.310	
Number	80		266		65	
Pseudo R-square	0.316		0.0942		0.184	
Log likelihood	-32.17		-166.90		-34.95	
Likelihood Ratio chi-square	29.78		34.74		15.72	

***p < 0.01, **p < 0.05, *p < 0.1.

## Discussion

Against the backdrop of urbanization, industrialization, and rural revitalization, the issues of non-grainization, non-agriculturalization, and anti-urbanization^54^ in rural households are becoming increasingly prominent, requiring managers to understand and guide them correctly. Especially in the hilly and mountainous areas of southern China, farmers are mostly risk-averse, and their livelihood transformation decisions not only seek to maximize profits but also consider minimizing risks.

**Fig. 7:**
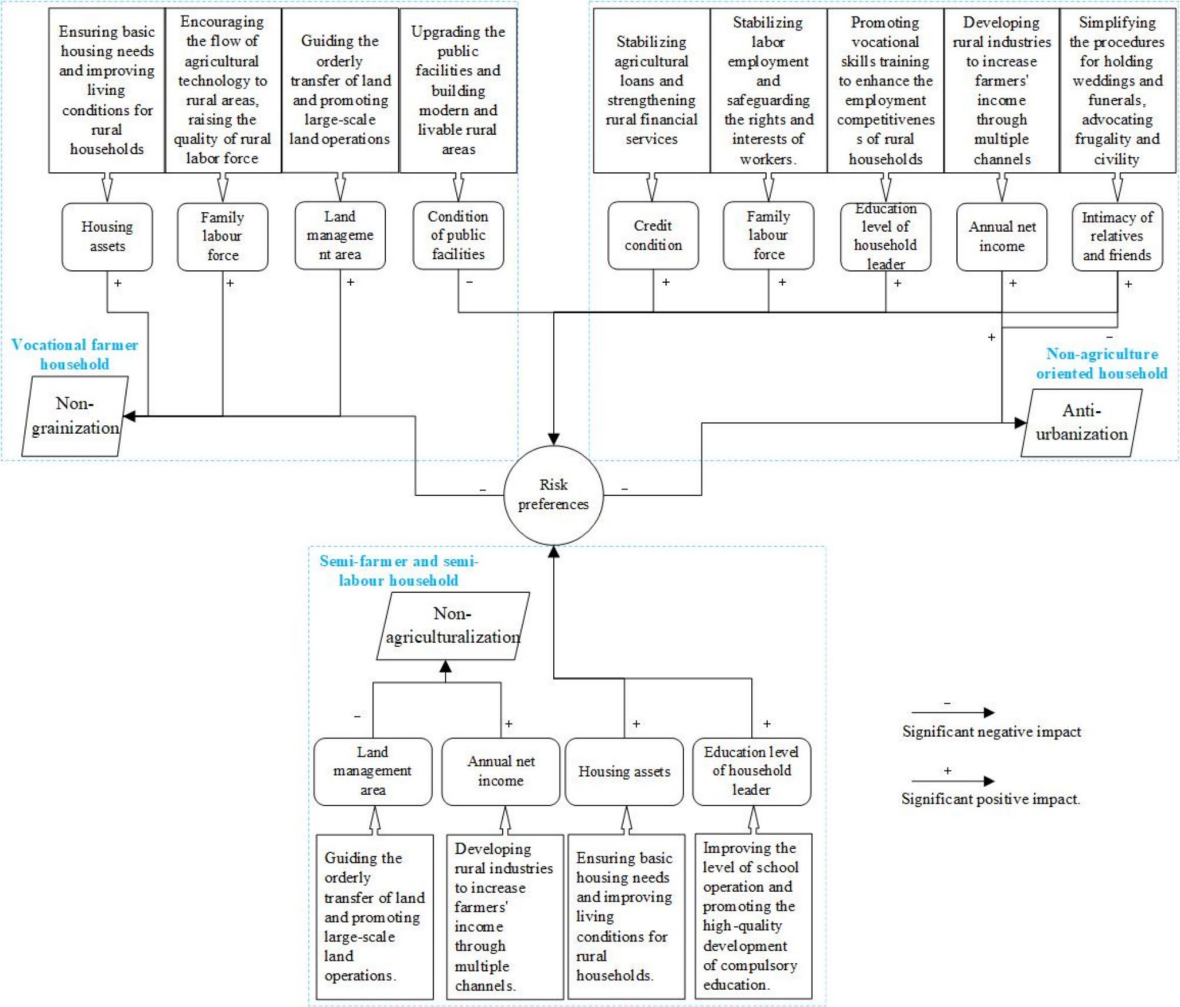
Rural livelihood transition mechanism and countermeasures.

For vocational farmer households in hilly and mountainous areas, where there are many mountains and limited farmland, high operating costs, and low grain production efficiency, planting special economic crops that are in line with the resource environment and cultivation conditions of mountainous areas canreduce risks and increase benefits. In the context of farmland protection and from the broader food perspective, apart from traditional grains like rice, it may be worthwhile to diversify the cultivation of characteristic crops in mountainous areas such as oil plants, vegetables, fruits, and tea. The government may need to establish a broader concept of food security and adjust crop structures, guide farmers to fully utilize mountainous forests, and make appropriate use of arable land^55^. Additionally, local government should increase investment in rural infrastructure and affordable housing construction, improve the accessibility of public services for vocational farmer households, and enhance their risk-bearing capacity. More efforts are needed to enhance rural social services, such as bringing agricultural technology to the countryside, providing vocational skills training, certifying land transfers, etc., to elevate the level of the family labour force and scale of land management for vocational farmer households (see Fig. 7).

For non-agriculture oriented households, despite obtaining relatively stable employment and income in the city, 51.1% of them still consider returning to their hometown for entrepreneurship or farming as a fallback livelihood. The anti-urbanization behavior of non-agriculture oriented households is primarily driven by nostalgia for their hometown, attachment to the land, or risk aversion. However, the current rural management lacks corresponding mechanisms and policy support, such as the imperfect system of returned village elites^56,57^, lagging public services^19^, unfriendly entrepreneurial environment^58^, and difficulties in financial support for rural areas^59^. Therefore, management authorities can explore promoting the system of local talents, innovating rural social governance through changing outdated customs, increasing investment in rural finance, and creating favorable conditions for non-agriculture oriented households to return to their hometowns.

For semi-farmer and semi-labour households, land assets and household income are key factors that affect their decision to move to the city or return to their hometown. The larger the land management area, the weaker the inclination toward non-agricultural transformation; the higher the household income, the stronger the inclination toward non-agricultural transformation. However, the existing dual urban-rural system^60^has created a large gap in education, health care, employment, and income welfare between urban and rural areas. Most rural households face the dilemma of being unable to enter the city or return to the countryside^61^. Therefore, managers should strengthen rural infrastructure construction, improve public services, develop characteristic advantages in rural industries, increase farmers’ income through multiple channels, and systematically promote rural institutional reforms to ensure that farmers can “go out and come back”.

## Conclusions

This study contributes to the literature on rural livelihood transition towards non-grainization, non-agriculturalization, and anti-urbanization based on a questionnaire survey of 411 typical households (vocational farmer household, semi-farmer and semi-labour household, non-agriculture oriented household) in a typical area (the hilly and mountainous region of southern China). It uses the Multiple Price List method and regression analysis to simulate the risk preferences and livelihood transition of rural households, highlighting the decision-making mechanism of non-agriculturalization, non-grainization, and anti-urbanization of rural households. The main conclusions are as follows:

76.86% of rural households in Ruijin city belong to the risk-averse type, of which 60.67% are highly risk averse. The ranking of risk aversion among these three typical types of households is consistent with the abundance of livelihood assets, in the order of non-agriculture oriented household > semi-farmer and semi-labour household > vocational farmer household. Among them, vocational farmer households are mainly suburban distributed and in the valley economic zone amid the mountains. This kind of farmer mainly grows characteristic crops, seasonal fruits, and vegetables, such as navel oranges, lotus seeds, and tobacco. Their main livelihood risks are pests and diseases, market fluctuations, and climate change. Semi-farmer and semi-labour households, as a dual-income livelihood, not only broaden their income sources but also effectively mitigate risks. They are pervasively scattered in the ecological functional zones and the valley economic zones, with their main livelihood risks being non-agricultural unemployment and agricultural disasters. Non-agriculture oriented households are mainly located in the urban development zone and the ecological functional zone, with their main livelihood risks being economic fluctuations, unemployment, and aging-related illnesses.

The decision of non-grainization among vocational farmer households is significantly and positively correlated with the family labour force, land management area, and housing assets, but negatively correlated with risk preference. It has formed a mature and stable industrial chain regarding the production, processing, and sales of local economic crops such as navel oranges, tobacco, and lotus seeds. The small-scale planting pattern of economic crops in mountainous areas (mainly navel oranges) and small farmland (mainly tobacco, and lotus seeds) fits the practical situation of mountainous area. Compared to growing grains, non-grain cultivation is less risky and more profitable. Therefore, when facing risks, 27.5% of vocational farmer households still choose to plant economic crops. Family labour force and land management area are the preconditions for vocational farming. While risk preference is negatively correlated with the condition of public facilities due to the perishability of agricultural products and their sensitivity to transportation distance.

Due to the risk-resilient characteristic of diversified livelihoods, the non-agricultural transition among semi-farmer and semi-labour households is not significantly related to their risk preferences, but negatively correlated with the land management area and positively correlated with annual net income. When risks occur, only 36.9% of semi-farmer and semi-labour households choose a complete transition to the non-agricultural sector. Improving the education level of household leaders and housing assets can effectively enhance the risk resilience of this kind farmer.

The anti-urbanization behavior of non-agriculture oriented households is significantly and negatively correlated with risk preferences and intimacy of relatives and friends, but positively correlated with annual net income. The higher the income level of non-agriculture oriented households, the stronger the desire to return to the countryside for health and leisure agriculture. Notably, excessive expenditures on interpersonal relationships have become a heavy burden and a major concern for non-agriculture oriented households returning to the countryside. When risks occur, 51.1% of non-agriculture oriented households choose to return to the countryside for entrepreneurship or agricultural work. Returning to countryside for agricultural work is still considered as a secure livelihood option by non-agriculture oriented households.

## Data Availability

The associated dataset of the study is available upon request to the corresponding author.
